# Synthesis and Properties of a Clean and Sustainable Deicing Additive for Asphalt Mixture

**DOI:** 10.1371/journal.pone.0115721

**Published:** 2015-01-27

**Authors:** Chao Peng, Jianying Yu, Zhijie Zhao, Jing Dai, Jingyi Fu, Meiling Zhao, Wei Wang

**Affiliations:** 1 State Key Laboratory of Silicate Materials for Architectures, Wuhan University of Technology, Wuhan, 430070, PR China; 2 Research Institute of Highway of Ministry of Transport, Beijing, 100088, PR China; 3 Key Laboratory of Advanced Technology for Special Functional Materials of Ministry of Education, Wuhan, 430070, PR China; University of Akron, UNITED STATES

## Abstract

A clean and sustainable deicing additive was prepared via the adsorption of acetate anions (Ac^-^) by magnesium (Mg) and aluminum (Al) calcined layered double hydroxide (Mg/Al-CLDH). Fourier transform infrared spectroscopy spectrums proved that Ac^-^ had intercalated into LDH structure. X-ray diffraction patterns, scanning electron microscopy and transmission electron microscopy images showed that the intercalation spacing and platelet thickness of Mg and Al layered double hydroxide containing Ac^-^ anions (Mg/Al-Ac^-^ LDH) had been enlarged due to substitution of divalent CO_3_^2-^ anions by a larger quantity of monovalent Ac^–^ anions. Differential scanning calorimetry tests testified that the insoluble Mg2/Al-Ac^-^ LDH evidently decreased the freeze point (FP) of water to -10.68°C. X-ray photoelectron spectroscopy analyses confirmed that the Ac- were strongly confined by the metal layers of LDHs. FP test of asphalt mixtures confirmed that Mg/Al-Ac^-^ LDHs reduced FP to -5.5°C. Immersion test results indicated that Mg/Al-Ac^-^ LDH had a good deicing durability and Ac^-^ did not released from asphalt mixture. Snow melting observation was conducted further testified that Mg/Al-Ac^-^ LDH melted snow or ice sustainably.

## Introduction

Ice may impose a potential risk for traffic safety, because tire skidding, brake failure or even losing control of direction are prone to take place on the pavement. Conventional prevention method is spraying chloride salts, which are including NaCl, CaCl_2_ or MgCl_2_. However, excessive use of chloride-based salt pollutes the surrounding environment and promotes corrosion in steel structure of vehicles, bridge and water pipes [1–5]. In addition, chloride anions would weaken the bonding strength between asphalt and aggregate, thereby affecting the service life of asphalt pavement [6, 7]. To mitigate these harmful effects, alternatives such as calcium-magnesium acetate (CMA) has been considered [8, 9]. Acetate-based salts have the similar melting snow or ice efficiency to that of chloride, and they are less harmful for the surrounding environment [10–12]. Nevertheless, it cannot melt snow or ice sustainably on the asphalt pavement, because rain or melted snow will wash the acetate anions (Ac^-^).

The method of incorporating the chloride deicing additives (Cl^-^DIA) in asphalt mixture has been developed since the last few decades [13–15]. Cl-DIA was added into asphalt mixture to play a role of self-melting snow or ice. Chloride component will gradually migrate from the interior structure of asphalt pavement due to the compression, vibration and wear from traffic load [16, 17]. However, the released chloride anions still pollute the surrounding environment and construction to some extent. Therefore, it is essential to develop a clean and durable deicing additive in the asphalt mixture.

Layered double hydroxides (LDHs) have attracted a great attention from many researchers owing to its anion exchangeable characteristic [18]. Its general formula of LDH is [M_1-x_^2+^M_x_^3+^·（OH）_2_]^x+^A_x/n_^n-^·mH_2_O, while M^2 +^ is a divalent metal cation, M^3 +^ is a trivalent metal cation, A^n-^ is an interlayer anion [19]. Previous papers have extensively reported that different types of anions can be intercalated into LDHs to inhibit or control their releasing process in aqueous solution [20–23]. In our preliminary test, we found that the freeze point of magnesium (Mg) and aluminum (Al) carbonate layered double hydroxide (Mg/Al-CO_3_^2-^ LDH) slurry is marginally lower than that of distilled water.

In this paper, magnesium (Mg) and aluminum (Al) acetate layered double hydroxide (Mg/Al-Ac^-^ LDH) was prepared from Mg/Al-CO_3_^2-^ LDH by calcination recovery. The structure, component, and morphology of Mg/Al-Ac^-^ LDH were characterized using Fourier transform infrared spectroscopy (FTIR), X-ray diffraction (XRD), scanning electron microscopy (SEM), transmission electron microscopy (TEM), and X-ray photoelectron spectroscopy (XPS). The effect of Mg/Al-Ac^-^ LDH on decreasing freezing point (FP) of water was identified by differential scanning calorimetry (DSC) tests. Then, Mg/Al-Ac^-^ LDH and Cl^-^DIA were added to asphalt mixtures to prepare Marshall specimen. The deicing properties of the mixtures were evaluated by FP measurement, immersion test and snow melting observation.

## Material and Methods

### Materials

The Mg/Al-CO_3_^2-^ LDH was provided by Jiangyin Ruilaw Chemical Co. LTD, Jiangsu, China. The structural formula of the respective LDH was Mg_1-x_Al_x_(OH)_2_(CO_3_)_2/x_·mH_2_O. The LDH samples were heated at 500°C for 4 h and the calcined Mg/Al-CO_3_^2-^ LDH sample is denoted as Mg/Al-CLDH. The physical and chemical properties of the samples are shown in Table 1. A commercial Cl^-^DIA was provided by Gezhouba Wuhan Road Materials Co.LTD, Hubei, China. Styrene—Butadiene—Styrene (SBS) modified asphalt that was produced by the China Best Modified Asphalt Co. LTD, Hubei, China and had a penetration of 48 dmm at 25°C, a ductility of 30 cm at 5°C, a softening point of 81°C, and a dynamic viscosity of 2.4 Pa.s at 135°C. Basalt and mineral filler (MF) (machine-glazed limestone) were obtained from Jingzhu, Hubei, China. All of the chemicals and reagents were analytical grade (Sinopharm Chemical Reagent Co., Ltd) and were used without further purification. Distilled water was used throughout the preparation and treatment processes.

**Table 1:** The physical and chemical properties of the Mg/Al-CLDHs.

**Sample**	**Molar ratio (Mg^2+^/Al^3+^)**	**Surface area(m^2^/g)**	**Average pore size(nm)**	**Pore volume(cm^3^g^-1^)**
Mg_2_/Al-CLDH	2:1	35.9	26.9	0.24
Mg_3_/Al-CLDH	3:1	33.5	25.6	0.22
Mg_4_/Al-CLDH	4:1	31.7	24.8	0.21

### Preparation of Mg/Al-Ac^-^ LDH

Mg/Al-Ac^-^ LDH was prepared from Mg/Al-CO_3_^2-^ LDH by calcination recovery. First, 200g Mg/Al-CO_3_^2-^ LDH was calcined at 500°C for 2 h in a muffle furnace to remove CO_3_^2-^ anions. The cooling process was operated in a closed stored pan, which had to be evacuated air immediately by a vacuum pump to avoid carbon dioxide. Second, the calcined product and 500 ml of potassium acetate solution (concentration of 30 wt%) were mixed in a glass flask and the mixture was stirred for 2 h. Third, the mixture was filtered and the residue was dried at 105°C for 24 h and was grinded to obtain Mg/Al-Ac^-^ LDH.

### Characterizations

The FTIR spectra of Mg_(2–4)_/Al-CO_3_^2-^ LDH and the Mg_(2–4)_/Al-Ac^-^ LDH were obtained using a Nexus FTIR spectrometer from Thermo Nicolet Corp. (New York, USA). The scan range was between 4000 cm^-1^ and 400 cm^-1^ with a resolution of 4 cm^-1^. XRD graphs of the Mg_(2–4)_/Al-CO_3_^2-^ LDH and the Mg_(2–4)_/Al-Ac^-^ LDH were obtained using an X-ray diffractometer (D/MX-III A, Rigaku, Japan) with Cu Kα radiation (λ=0.15406 nm; 40 kV, 50 mA). The data were collected in step-scan mode at a scanning rate of 0.02°/s. The diffractograms were scanned from 5° to 20°. The freezing points of the 5 wt% slurries of Mg_2_/Al-Ac^-^ LDH and Mg_2_/Al-CO_3_^2-^ LDH were determined using a DSC thermal analysis system (Perkin-Elmer 7 Series) that was equipped with a cooling accessory. Before recording the warming scans, a 5-mg slurry sample was sealed in an aluminum pan, cooled to -40°C in a nitrogen atmosphere and then heated to 20°C at a rate of 2°C /min. The freezing point of LDH slurry corresponds to the onset temperature of the DSC curve, and the peak area corresponds to the enthalpy of adsorption. The Mg_2_/Al-CO_3_^2-^ LDH and Mg_2_/Al-Ac^-^ LDH powders were attached to stubs using a double-sided adhesive (carbon tape). The electrical conductivities of the samples were enhanced by sputtering the sample surfaces with a thin film of platinum using a sputter coating system (Baltec MED020, Vaduz, Liechtenstein). The sample morphologies were investigated by SEM (Quanta 250 FEG, FEI, Oregon, USA). TEM analyses were conducted using a JEM-2100F electron microscope (JEOL, Japan) at a 200-kV accelerating voltage.

### Preparation of asphalt mixture with deicing additives

**Figure 1:**
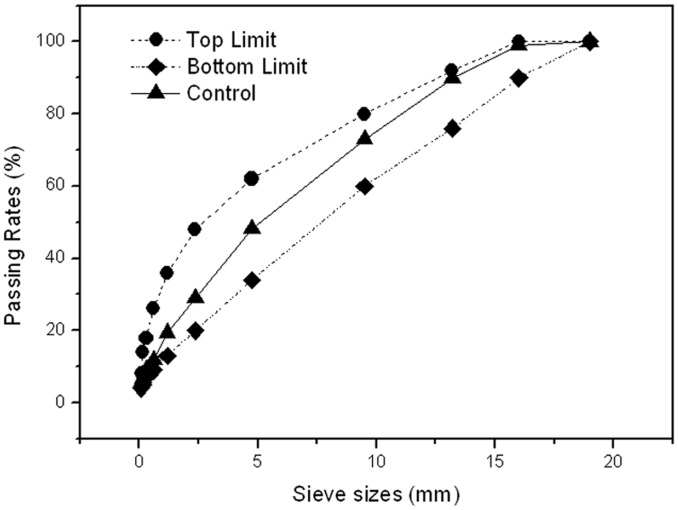
The gradation of the control mixture.

An AC-16 asphalt mixture is commonly used in the surface layer of asphalt pavement. According to the standard JTG F40–2004 (Technical Specification for Construction of Highway Asphalt Pavements, the Ministry of Transport of the People’s Republic of China), designed gradation of control mixture containing 6wt% MF is shown in Fig. 1.

The optimum asphalt content was determined to be 5.3 wt% from the Marshall test. The particle size distributions of the deicing additives (Mg_2_/Al-Ac^-^ LDH and Cl^-^DIA) and the MF were given in Table 2. The particle sizes and density of Mg_2_/Al-Ac^-^ LDH and Cl^-^DIA were similar to that of MF. Thus, in this experiment, the asphalt mixture was prepared by replacing the MF with equal weights of the two deicing additives.

**Table 2:** Particle size distribution of deicing additives and mineral filler.

**Sieve Size/mm**	**Passing Rate/%**				
	**1.18**	**0.6**	**0.3**	**0.15**	**0.075**
Mg_2_/Al-Ac^-^ LDH	100	94.56	87.54	61.33	39.35
Cl^-^DIA	100	96.32	86.24	53.27	36.49
MF	100	97.58	88.94	62.79	43.56

### Specimen preparation

The SBS modified asphalt was mixed with aggregates at 160°C for 90 s to obtain a well-distributed mixture. The Cl^-^DIA and MF were subsequently added to mixtures and mixed for 60 s. The hot mixtures were then placed in steel frames and compacted using 75 blows for each surface at 150°C to obtain the Marshall specimens (Φ101.6 mm×63.5 mm).

### Evaluation of deicing properties of asphalt mixture

**Figure 2:**
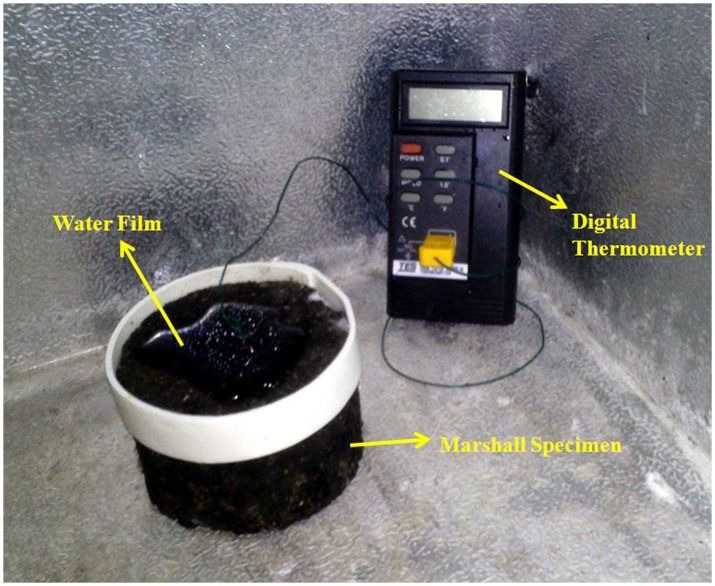
Scene picture for testing the FP of a Marshall specimen in a refrigerator.

**Freezing points of asphalt mixtures**. Deicing additives are used to reduce the FPs of asphalt pavements. Therefore, it is essential to measure the FP of an asphalt mixture to evaluate the effect of deicing additive. The testing temperature range was from 5°C to -10°C. The following test method was used to determine the FPs. First, the Marshall specimen was placed in a refrigerator at 5°C for 1 h to achieve an equivalent temperature with environmental temperature. Then, 10 g of distilled water was added on the surface of the Marshall specimen and a plastic ring was firmly pasted around the edge by silicone adhesive to seal the water. A thermocouple with a precision of 0.1°C (TES-1310, by TES Electrical Electronic Co., LTD in Taiwan) was used to automatically record the temperature of the water film every 10 s with a data logger. The reflection peak point of output time-temperature curve was considered as the FP. Fig. 2 illustrates the scence picture for testing the FP of a Marshall specimen.

**Figure 3:**
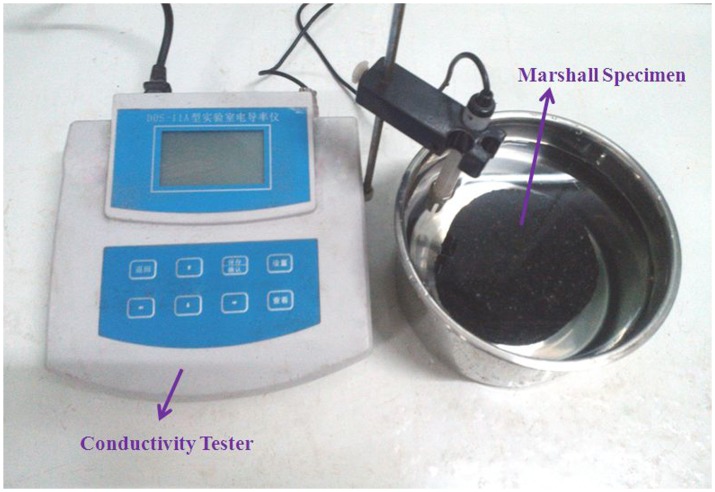
Scene picture of measuring the conductivity of immersion test.

**Conductivity of immersion liquid**. The conductivity of a liquid increases with the ion concentration [24], thus, the rate of ion release from a deicing asphalt mixture was determined by measuring the conductivity of the immersed liquid at different periods. The following measurement procedure was used. A Marshall specimen was completely immersed in 500 ml of deionized water at 25±1°C in a stainless steel container. The conductivity of the immersed liquid was then measured by a conductivity tester (DDS-11C, by Jingke Scientific Instrument Co., LTD in Shanghai, China) every 24 h and the immersed liquid was completely substituted with fresh water each day to simulate the washing cycles of real melted snow or rain. In addition, pavement abrasion caused by traffic load is considered as another critical factor to affect the amount of released ions. A mechanical abrasion by steel bush was applied on the Marshall specimen surface at a 5-days interval and the immersion test was conducted for a couple of days. Fig. 3 illustrates the scene picture of conductivity test.

**Freezing point of asphalt mixture after immersion**. To evaluate the effect of rain on the ice-melting performance of an asphalt pavement that contains a deicing additive, the FPs of asphalt mixtures were measured after each immersion cycle.

## Results and Discussion

### FTIR

**Figure 4:**
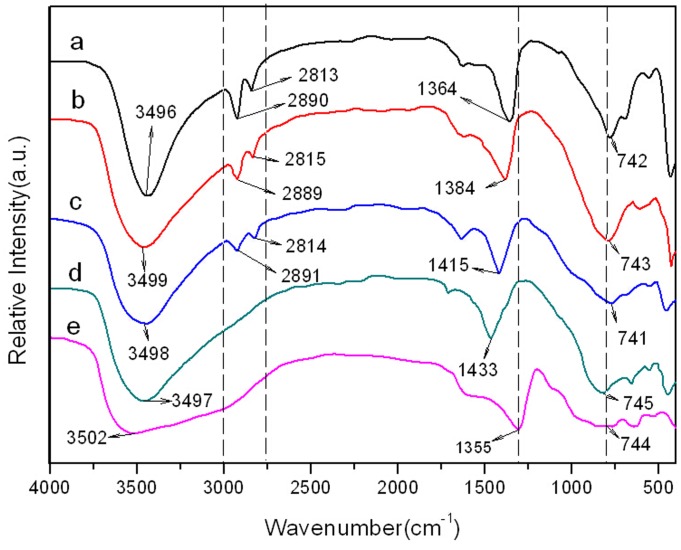
FT-IR spectra. (a) Mg_2_/Al-Ac^-^ CLDH, (b) Mg_3_/Al-Ac^-^ CLDH, (c) Mg_4_/Al-Ac^-^CLDH, (d) Mg_2_/Al-CLDH, (e) Mg_2_/Al-CO_3_^2-^ LDH.

FTIR was used to identify whether Ac^-^ anions have intercalated in the Mg/Al-CLDH. Fig. 4a, b and c show that the Mg_(2–4)_/Al-Ac^-^ LDH samples exhibit two bands between 2750 and 3000 cm^−1^ assigned to the vibrational absorption of the methyl group, while Fig. 4d and e do not show any characteristic peaks in this range. It indicated that acetate anions (Ac^-^) had entered the LDH interlayer galleries of Mg_(2–4)_/Al-Ac^-^ LDH. It is noteworthy that the intensities of the methyl peaks increased as the Mg/Al molar ratio decreased. This is because Mg_2_/Al-CLDH with the higher surface area than Mg_3_/Al-CLDH and Mg_4_/Al-CLDH (see in Table 1) can absorb larger amount of acetate anions. The peak at 1355 cm^−1^ in the spectrum of the Mg_2_/Al-CO_3_^2-^ LDH sample was attributed to the vibrational absorption of the interlayer CO_3_^2-^, as reported previously [25]. After the calcination and adsorption of Ac^-^, this band shifted to a higher wavenumber. This is because a small amount of carbon dioxide has been absorbed by wet Mg_(2–4)_/Al-Ac^-^ LDH during the drying process [26, 27]. For all FTIR spectra, the peaks appeared between 400 and 800 cm^−1^ were arisen from a superposition of the characteristic vibrations of Mg and Al oxides and the wide and extremely intense peaks at approximately 3500 cm^−1^ were attributed to hydrogen bonds.

### XRD

**Figure 5:**
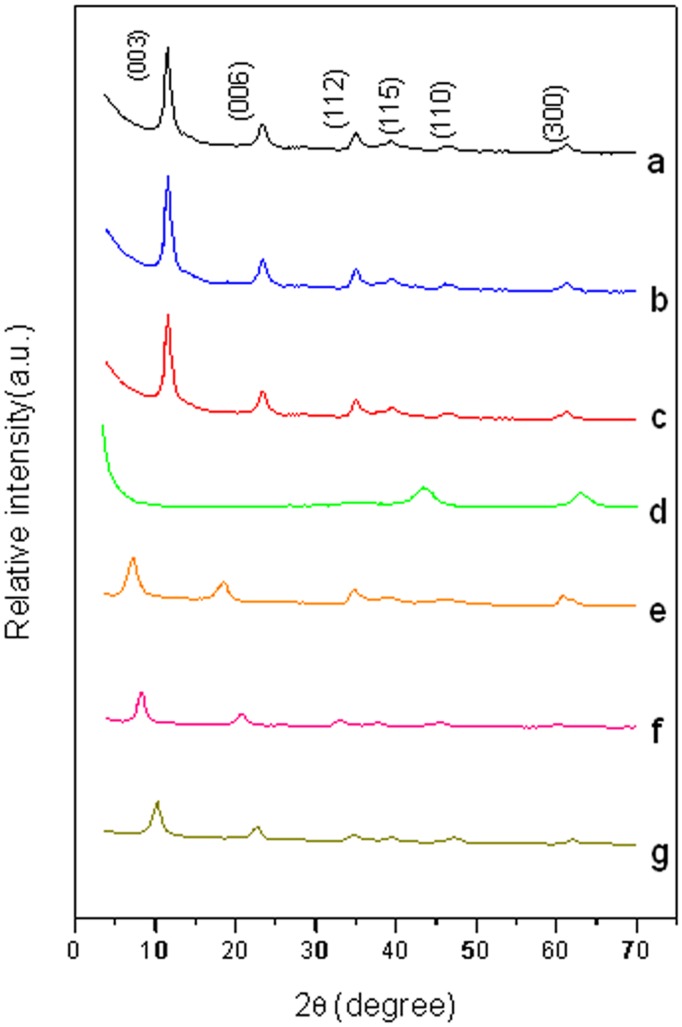
XRD patterns. (**a**) Mg_2_/Al-CO_3_^2-^ LDH, (b) Mg_3_/Al-CO_3_^2-^ LDH, (c) Mg_4_/Al-CO_3_^2-^ LDH, (d) Mg_2_/Al-LDH, (e) Mg_2_/Al-Ac^-^ LDH, (f) Mg_3_/Al-Ac^-^ LDH, (g) Mg_3_/Al-Ac^-^ LDH.

XRD can be used to analyze the crystal structure of Mg_(2–4)_/Al-CO_3_^2-^ LDH, Mg_2_/Al-CLDH and Mg_(2–4)_/Al-Ac^-^ LDH. Fig. 5 (a, b, c) show that Mg_(2–4)_/Al-CO_3_^2-^ LDH have a series of sharp and strong diffraction peaks（003 and 006）at low 2θ values and small symmetric peaks (112, 115, 110 and 300) at high 2θ values. These peaks are typical reflections of the LDH metal layers. The interlayer distances of d (003) for Mg_(2–4)_/Al- CO_3_^2-^ LDH are both 7.56 Å, which can be calculated by the Bragg formula (nλ=2dsinθ). In this formula, n is an integer (n = 1), λ is the wavelength of incident wave (1.54nm) and d is the interlayer spacing. After calcination, the diffraction peaks (003 and 006) at a low 2θ values disappear and the peak area ratios are also changed (Fig. 5d), indicating that the layered structure is destroyed during calcinations. Fig. 5 e, f, and g reveal that (003), (006) and (112) diffraction peaks appeared again after calcinations. That means that Mg_(2–4)_/Al Ac^-^ LDH have restored their layered structures after the adsorption of Ac^-^ due to the “memory effect”. In addition, it can also be seen that the 2θ values of (003) peaks for Mg_(2–4)_/Al-Ac^-^ LDH are 6.41, 9.12 and 10.63, corresponding to the interlayer distances of 13.77 Å, 9.69 Å and 8.32 Å. This result could be attributed to varying amount of Ac^-^ among the metal cation layers, which are composed of divalent magnesium cations and trivalent aluminum cations. When the Mg/Al molar ratio decreased, the proportion of Al^3+^ increased and therefore the LDH metal cation layers can absorb more number of monovalent Ac^-^. As a result, the interlayer distance of LDH will be enlarged due to the increased amount of Ac^-^, which is in accordance with FTIR analysis. Further observation on the weakened intensity of the Mg_(2–4)_/Al-CLDH diffraction peaks after uptake of Ac^-^ revealed that the crystallinity is decreased. This is owing to the calcination and rehydration procedure [28]. The diffraction angles and interlayer distances of LDHs are shown in Table 3.

**Table 3:** Diffraction angles at peak (003) and interlayer distances of LDHs.

**Specimen**	**Mg_2_/Al CO_3_^2-^LDH**	**Mg_3_/Al CO_3_^2-^LDH**	**Mg_4_/Al CO_3_^2-^LDH**	**Mg_2_/Al-CLDH**	**Mg_2_/Al Ac^-^ LDH**	**Mg_3_/Al Ac^-^ LDH**	**Mg_4_/Al Ac^-^ LDH**
2θ (degree)	11.69	11.69	11.69	─	6.41	9.12	10.63
Interlayer Distance(Å)	7.56	7.56	7.56	─	13.77	9.69	8.32

### DSC

**Figure 6:**
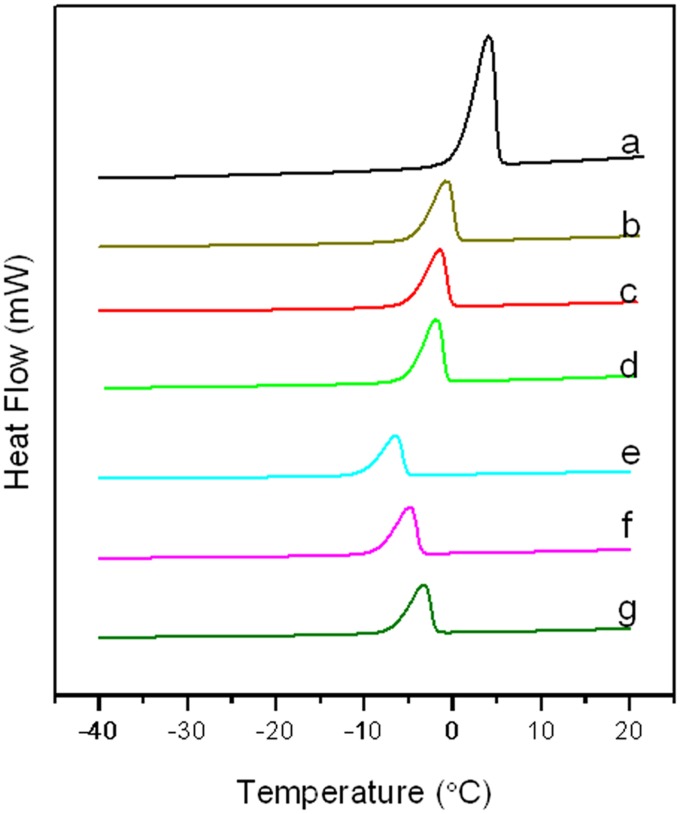
DSC curves. (**a**) pure water, (**b**) Mg_2_/Al-CO_3_^2-^ LDH, (**c**) Mg_3_/Al-CO_3_^2-^ LDH, (**d**) Mg_4_/Al-CO_3_^2-^ LDH, (**e**) Mg_2_/Al-Ac^-^ LDH, (**f**) Mg_3_/Al-Ac^-^ LDH, (**g**) Mg_4_/Al-Ac^-^ LDH.

To indentify the effect of Mg_2_/Al-Ac^-^ LDH and Mg_(2–4)_/Al-CO_3_^2-^ LDH on decreasing the FP of water, DSC was used to measure the FPs of their slurries. Fig. 6(b, c, and d) show that the FPs of the Mg_(2–4)_/Al-CO_3_^2-^ LDH of -3.48°C, -3.39°C, and -3.26°C were less than that of pure water (-2.53×10^-2^°C). Moreover, the endothermic peak areas (i.e., the enthalpy) of the Mg_(2–4)_/Al-CO_3_^2-^ LDH were 287.53 J/g, 293.47 J/g, and 298.76 J/g, which were less than that of pure water (375.68 J/g). That is, less heat was required to melt ice containing Mg_(2–4)_/Al-CO_3_^2-^ LDH powder than pure ice. These results indicate that Mg_(2–4)_//Al-CO_3_^2-^ LDH can marginally decrease the FP of water. Fig. 6 (e, f, and g) show that the FPs of the Mg_(2–4)_/Al-Ac^-^ LDH decreased to -10.68°C, -8.98°C, and -7.13°C, and the corresponding enthalpies decreased to 107.36 J/g, 121.39 J/g, and 130.58 J/g. It is attributed to the increased amount of the acetate anions in the LDH metal layers, which is consistent with FTIR and XRD analysis. This result confirms that Mg_2_/Al-Ac^-^ LDH has the better deicing capacity than the other two LDH deicers. The FPs and enthalpies of water and LDHs slurries are listed in Table 4.

**Table 4:** Freeze points and enthalpies of water and LDHs slurries.

**Specimen**	**pure water**	**Mg_2_/Al CO_3_^2-^LDH**	**Mg_3_/Al CO_3_^2-^LDH**	**Mg_4_/Al CO_3_^2-^LDH**	**Mg_2_/Al Ac^-^ LDH**	**Mg_3_/Al Ac^-^ LDH**	**Mg_4_/Al Ac^-^ LDH**
Freeze Point(°C)	-2.53×10^-2^	-3.48	-3.39	-3.26	-10.68	-8.98	-7.13
Enthalpy(J/g)	375.68	287.53	293.47	298.76	107.36	121.39	130.58

### Morphology

**Figure 7:**
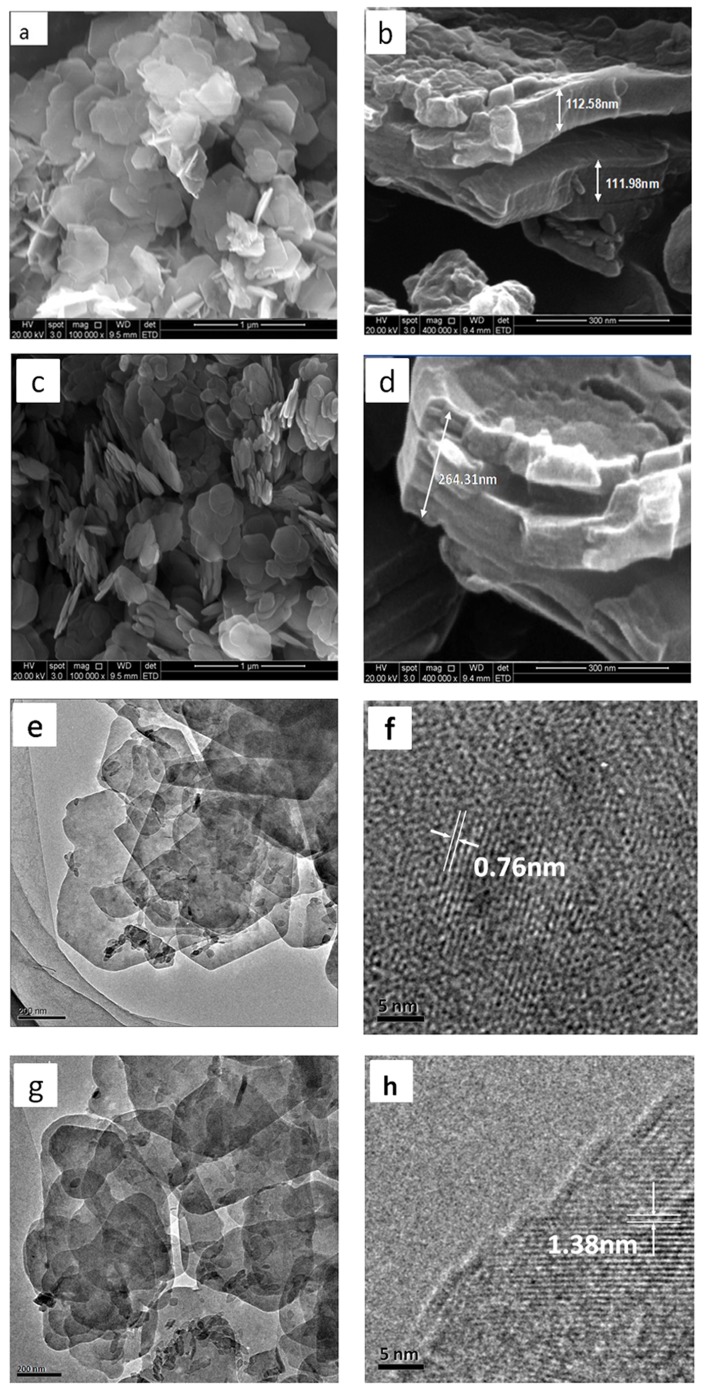
SEM and TEM images of the samples. Mg_2_/l- CO_3_^2-^ LDH (a, b), Mg_2_/Al-Ac^-^ LDH (c, d); TEM and high-resolution TEM images of the samples: Mg_2_/Al- CO_3_^2-^ LDH (e, f), Mg_2_/Al-Ac^-^ LDH (g, h).

The SEM photomicrographs for Mg_2_/Al-LDH and Mg_2_/Al-Ac^-^ LDH are shown in Fig. 7. Fig. 7a depicts that the Mg_2_/Al-CO_3_^2-^ LDH crystallites consisted of homogeneous platelets and that the crystallites dimension ranged from 200 nm to 500 nm. Fig. 7a and c show that the distribution of Mg_2_/Al-Ac^-^ LDH platelets was less uniform than that of the Mg_2_/Al-CO_3_^2-^ LDH platelets. This difference could be attributed to the decarbonation of Mg/Al-CO_3_^2-^ LDH during calcinations [29]. The release of CO_3_^2-^ anions could lead to the collapse of some metal cation layers, thereby rearranging the platelets. The “memory effect” of LDH [30] can be used to recovered Mg_2_/Al-Ac^-^ LDH platelets by the intercalation of Ac^-^ anions, which leads to defects and interconnected edges. Fig. 7b and d show that the thickness of each Mg/Al-CO_3_^2-^ LDH platelets was approximately 110 nm and that the thickness of the Mg_2_/Al-Ac^-^ LDH platelets was about 172 nm. The crystal platelets were stacked with numerous metal cation layers; thus, the increase in the Mg_2_/Al-Ac^-^ LDH platelet thickness was attributed to the enlarged interlayer distance among the metal cation layers. Fig. 7e confirms that the LDH sample is composed of hexagonal nanosheets with smooth surface, in agreement with the previous results [31]. Fig. 7f shows that the presence of an interlayer distance of about 0.76 nm（7.6 Å） is consistent with the distance of the (003) plane, corresponding with the X-ray analysis. It is noteworthy that the CLDH sample (Fig. 7g) nearly remains in the laminar shape of nanosheets with less regular shape and Fig. 7h further confirms that the intercalation of Ac^-^ has enlarged the interlayer distance of Mg_2_/Al-Ac^-^ LDH.

### XPS

**Figure 8:**
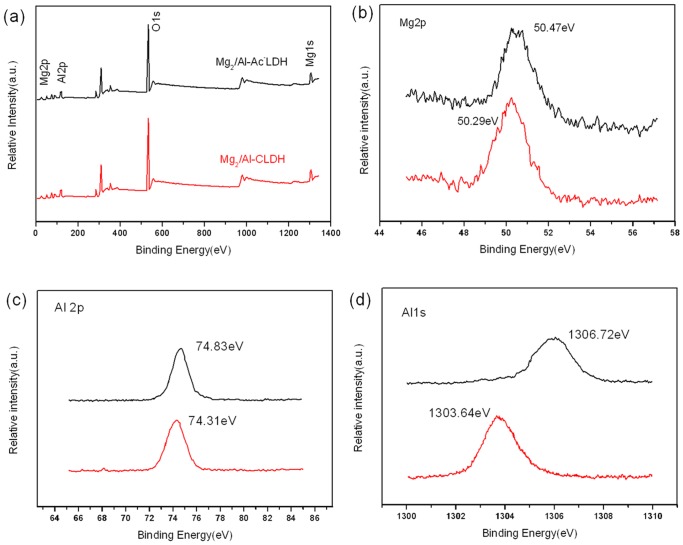
XPS survey spectra. (a) and high-resolution XPS spectra o f Mg 2p (b), Al 2p (c) and Al 1s (d) of Mg_2_/Al-LDH before and after adsorption of Ac^-^.

To indentify whether Ac^-^ anions have been confined in the Mg_2_/Al-Ac^-^ LDH, XPS was used to measure the binding energy of metal elements in Mg_2_/Al-CLDH and Mg_2_/Al-Ac^-^ LDH. Fig. 8a shows the XPS survey spectra of the Mg_2_/Al-CLDH samples before and after the adsorption of Ac^-^. The peaks at binding energies of 50.29, 74.31, and 1303.64 eV were related to Mg 2p, Al 2p, and Al 1s before the adsorption of Ac^-^, respectively. Fig. 8b shows that the binding energy of Mg 2p increased from 50.29 eV before adsorption to 50.47 eV after adsorption, indicating that the interaction between the Mg cations and the acetate anions was strengthened. Fig. 8c and d show that the binding energies of Al 2p and Al 1p increased from 74.31 eV and 1303.64 eV to 74.83 eV and 1306.72 eV. That means the electronegativity of Mg_2_/Al-Ac^-^ LDH is stronger than that of Mg_2_/Al-CLDH [28, 32, 33]. These positive shifts provided further evidence of the strengthening of the interactions between the Al cations and the acetate anions by adsorption. The aforementioned results show that the acetate anions entered into the host layer lattice of the Mg_2_/Al-CLDH during the rehydration process, and the anion interaction has a strong restricted effect on the motion of the acetate anions. Therefore, Mg_2_/Al-Ac^-^ LDH can produce a sustainable decrease in the freezing point of water.

### Evaluation of the deicing properties of asphalt mixtures

**Figure 9:**
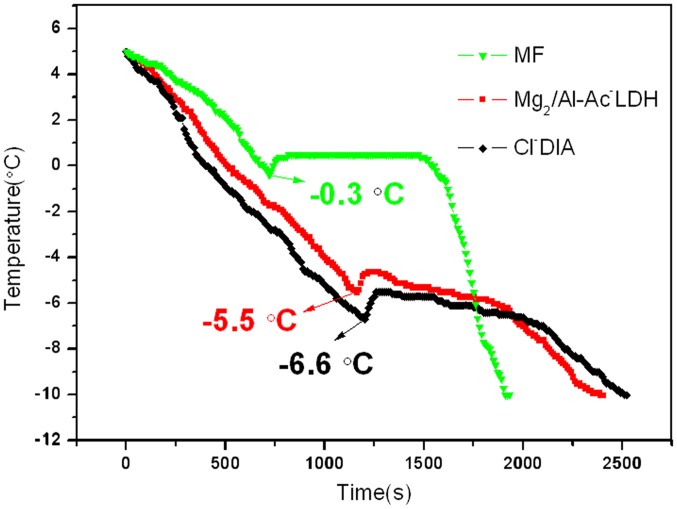
Freezing points of asphalt mixtures containing deicing additives.

**Freezing point of asphalt mixtures with deicing additives**. FPs of asphalt mixtures with MF, Mg_2_/Al-Ac^-^ LDH and Cl^-^DIA are shown in Fig. 9. Due to the supercooling process of water, there are both temperature reflection peaks in these curves [34]. In the MF group, asphalt mixture containing MF, the temperature of water film on the surface of MF asphalt mixture achieved the temperature reflection peak at -0.3°C after 720 s. Subsequently, a temperature plateau appeared near 0°C and eventually dropped down with prolonging time. In Mg_2_/Al-Ac^-^ LDH and Cl^-^DIA groups, temperature of water film on the surface of Mg_2_/Al-Ac^-^ LDH and Cl^-^DIA asphalt mixtures reduced to -5.5°C at 1150 s and -6.6°C at 1230 s, respectively. After then, the curves did not show a plateau but temperature kept going down. This can be attributed to that the FP of a liquid layer at the interface between asphalt mixture and ice continues to decrease because of decreasing volume of free liquid. Comparing these two freeze process, it can be found that FPs of Mg_2_/Al-Ac^-^ LDH and Cl^-^DIA groups were apparently lower than that of MF group. Moreover, freeze process of Mg_2_/Al-Ac^-^ LDH and Cl^-^DIA groups can be delayed. From the FP test results of asphalt mixtures containing Mg_2_/Al-Ac^-^ LDH and Cl^-^DIA and MF, it testifies that Mg_2_/Al-Ac^-^ LDH and Cl^-^DIA can both decrease the FPs of water films on the asphalt pavement and delay the freeze process.

**Figure 10:**
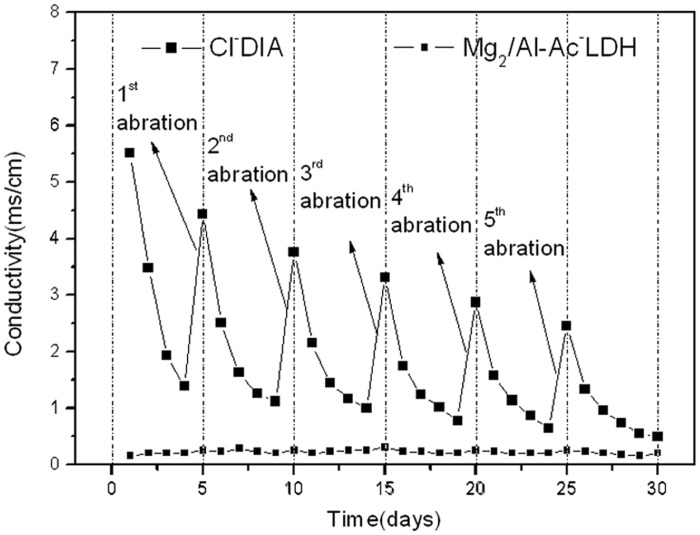
Conductivities of the immersion liquids of Marshall specimens containing deicing additives.

**Immersion liquid conductivity**. The conductivities of the immersion liquids for the Marshall specimen at different immersion times are shown in Fig. 10. After one day of immersion, the conductivity of the immersion liquid for the Mg_2_/Al-Ac^-^ LDH and Cl^-^DIA groups were 0.31 ms/cm and 5.56 ms/cm, implying that ions were well confined in the Mg_2_/Al-Ac^-^ LDH layered structure during the immersion. In the next three days, the conductivity of the immersion liquid for the Marshall specimen containing Mg_2_/Al-Ac^-^ LDH remained nearly constant (0.3 ms/cm) but the conductivities of the immersed liquid for the Marshall specimen containing Cl^-^DIA were gradually decreased. It indicated that Mg_2_/Al-Ac^-^ LDH had a good durability of fixing ions and the ions from Cl^-^DIA at the exterior of asphalt mixture was consuming. When the first mechanical abrasion was applied on the surface of asphalt mixture, the conductivity of immersion liquid for Cl^-^DIA group surged to 4.48 ms/cm but the conductivity of immersion liquid for Mg_2_/Al-Ac^-^ LDH group was still unchanged. In addition, the conductivities of immersion liquids for the Cl^-^DIA group showed a declining zigzag trend in the following mechanical abrasion cycles due to decreasing residual salt in asphalt mixture. These results implied that Ac^-^ anions in Mg_2_/Al-Ac^-^ LDH were not washed under dynamic conditions and the amount of releasing ions from Cl^-^DIA was gradually decreased.

**Figure 11:**
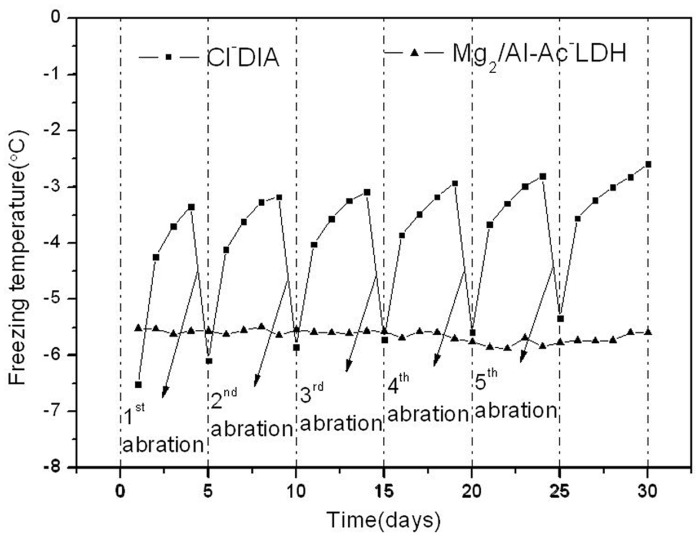
Freezing points of Marshall specimens after immersion.

**Freezing points of asphalt mixtures after immersion**. FPs for the asphalt mixtures with Mg_2_/Al-Ac^-^ LDH or Cl^-^DIA after immersion are shown in Fig. 11. The FP of the asphalt mixture with Cl^-^DIA increased from -6.5°C to -2.9°C after 4 days of immersion, showing that the deicing property of the Cl^-^DIA Marshall specimen had been significantly weakened. After the first mechanical abrasion, the FPs of Cl^-^DIA showed an increasing zigzag trend in the following mechanical abrasion cycles. It was interesting to underline that the freezing temperature of Mg_2_/Al-Ac^-^ LDH and Cl^-^DIA asphalt mixture after the 4^th^ mechanical abrasion were -5.5°C and -5.4°C, respectively. That indicated that the Mg_2_/Al-Ac^-^ LDH had better deicing capability than Cl^-^DIA after a certain degree of mechanical abrasion. In contrast, the FP of the Marshall specimen containing Mg_2_/Al-Ac^-^ LDH remained at around -5.5°C throughout the entire immersion process, implying that the deicing property of the asphalt mixture containing Mg_2_/Al-Ac^-^ LDH did not change during the long time immersion. The above results testified that Mg_2_/Al-Ac^-^ LDH had the sustainable anti-ice property.

**Figure 12:**
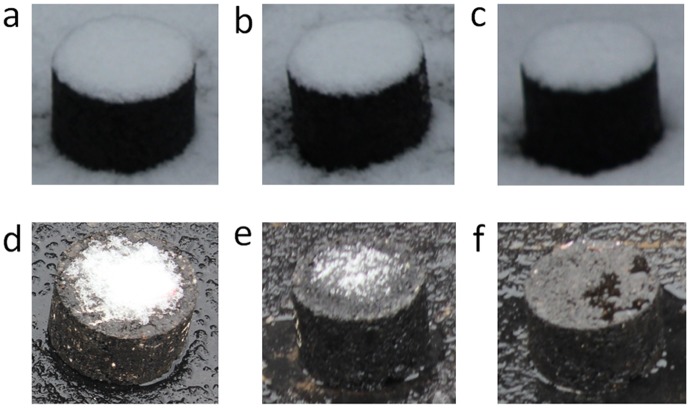
Photos of the first snow melting observation. (a). Asphalt mixture containing MF, (b). Asphalt mixture containing Mg_2_/Al-Ac^-^ LDH, (c). Asphalt mixture containing Cl^-^DIA; (d). Asphalt mixture containing MF after 4 h, (e). Asphalt mixture containing Mg_2_/Al-Ac^-^ LDH after 4 h, (f). Asphalt mixture containing Cl^-^DIA after 4 h.

**Figure 13:**
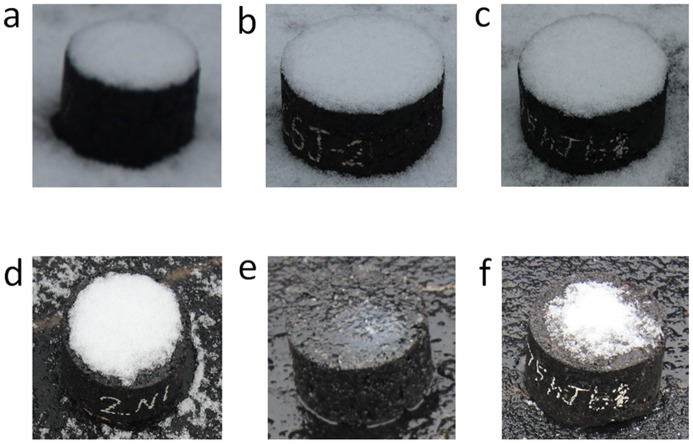
Photos of the second snow melting observation. (a). Asphalt mixture containing MF, (b). Asphalt mixture containing Mg_2_/Al-Ac^-^ LDH, (c). Asphalt mixture containing Cl^-^DIA; (d). Asphalt mixture containing MF after 4 h, (e). Asphalt mixture containing Mg_2_/Al-Ac^-^ LDH after 4 h, (f). Asphalt mixture containing Cl^-^DIA after 4 h.

**Snow melting observations**. As is known, melting accumulated snow can effectively prohibit the icy road surface. In this test, Marshall specimens were placed outdoors before the snow. The first snow event occurred on the night of 6^th^, February in 2014 and the temperature was about -4〜-5°C during the observation process. Photos of the first snow melting observation are shown in Fig. 12. Obviously, the accumulated snow on surface of asphalt mixture containing Mg_2_/Al-Ac^-^LDH and Cl^-^DIA nearly melted. It testified that the Mg_2_/Al-Ac^-^ LDH and Cl^-^DIA can melt snow in a short term. After that, the melted snow and rain washed the asphalt mixture several times in the next couple of days. The second snow event occurred on the morning of 12^th^, February in 2014 and the snow melting temperature was about -3〜-4°C. Photos of the second snow melting observation are shown in Fig. 13. It can be seen that asphalt mixture containing Mg_2_/Al-Ac^-^ LDH melted snow evidently but the asphalt mixture containing Cl^-^DIA melted less snow than the former. This result can be explained by that the exposed Cl^-^DIA had been solved by melt snow or rain but the insoluble Mg_2_/Al-Ac^-^ LDH can continuously play a role of melting snow. In practical asphalt pavement, traffic load is another key factor to melt snow or ice. Cl^-^DIA on the asphalt pavement will not be exhausted, because residual Cl^-^DIA from the interior layer of asphalt pavement keeps migrating to the road surface due to the mechanical abrasion. In other words, this static snow melting observation can only be used as an indirect approach to compare the effect of the melted snow or rain on melting-snow property of Mg_2_/Al-Ac^-^ LDH and Cl^-^DIA.

## Conclusions

On the basis of the characterizations of Mg_2_/Al-Ac^-^ LDH and its deicing property, the main findings and conclusions can be drawn.

(a) FTIR spectra confirmed that Ac^-^ had intercalated into Mg-Al LDH structure. XRD patterns showed that the interlayer distance of Mg_2_/Al-Ac^-^ LDH was larger than those of Mg/Al-CO_3_^2-^ LDH and other Mg_(3,4)_/Al-Ac^-^ LDHs, implying that a larger quantity of monovalent Ac^-^ anions was required to compensate for the lost divalent CO_3_^2-^ anions. DSC curves demonstrated that Mg_2_/Al-Ac^-^ LDH significantly reduced the FP of water. SEM showed that the Mg_2_/Al-Ac^-^ LDH platelets were thicker than that the of Mg_2_/Al-CO_3_^2-^ LDH platelets and TEM further confirmed that the interlayer distance of Mg_2_/Al-Ac^-^ LDH had been enlarged due to that double-layer distance of monovalent Ac^-^ anions was wider than single-layer distance of divalent CO_3_^2-^ anions. XPS spectra proved that Ac^-^ anions were strongly confined in LDH structure.

(b) The conductivity of the immersion liquid for the Marshall specimen showed that Mg_2_/Al-Ac^-^ LDH constrained the Ac^-^ anions in the Marshall specimens. FPs of the asphalt mixtures indicated that Mg_2_/Al-Ac^-^ LDH had a better anti-ice durability than Cl^-^DIA. Snow melting observation further testified that Mg_2_/Al-Ac^-^ LDH sustainably melted snow or ice and did not pollute environment.
